# Local Anesthesia

**Published:** 1899-10

**Authors:** 


### Local Anesthesia.

Dr. Bagot (*Journal des Practiciens*) considers that the tonic action of sparteine on the heart combats the depressant effect of cocaine, while aiding the local anesthetic effect of the latter. He recommends the following in each powder:

Hydrochloride of cocaine, - - $\frac{1}{2}$ grain.

Sulphate of sparteine, - - - - $\frac{3}{4}$ grain.

For use, dissolve one powder in either fifteen or thirty drops of boiled water at the time of injection. Begin with the weaker solution. Fifteen drops are injected in the line of operation on one side of the small tumor to be removed; after waiting seven or eight minutes, the other half on the other side. At the end of some moments he commences the operation on the side first injected, and by the time that is finished anesthesia is complete on the other side.—*N. Y. Med. Journal.*

### Earache.

Atropine, in solution, is an excellent remedy for earache. A drop or two of a solution of four grains to the ounce of water will be sufficient.—*Med. Review of Reviews.*

